# Supplementary material for: Trichoderma atroviride Enhances Impatiens walleriana Hook. f Growth and Flowering in Different Growing Media
Source: Plants (Basel). 2024 Feb 21;13(5):583. doi: 10.3390/plants13050583 (PMC10934068; doi:10.3390/plants13050583)
Supplement: Supplementary file 1 [file plants-13-00583-s001.zip › plants-2859661-supplementary.pdf]

Supplementary data

Trichoderma atroviride enhances *Impatiens walleriana* Hook. f growth and flowering in different growing media

Silvia Traversari[1,2], Mariateresa Cardarelli[3*], Massimo Brambilla[4], Maurizio Cutini[4], Gianluca Burchi[5], Marco Fedrizzi[6], Daniele Massa[5], Alessandro Orlandini[6,7], Sonia Cacini[5]

Table S1 Biometric parameters of *I. walleriana* plants at the end of the experiment.

Source of variation	Flower DW (g plant^{-1})	SLA (cm^2 g DW^{-1})	Root/Shoot
Treatment (T)			
No *T. atroviride* (NTA)	0.87 ± 0.278	565 ± 83.8	0.24 ± 0.045
T. atroviride (TA)	1.08 ± 0.121	606 ± 74.5	0.23 ± 0.034
P-value	ns	ns	ns
Substrate (S)			
Peat (P)	1.03 ± 0.190	565 ± 95.9	0.25 ± 0.047
Coir (C)	0.92 ± 0.273	606 ± 58.6	0.22 ± 0.022
P-value	ns	ns	ns
T × S			
NTA × P	0.97 ± 0.243	553 ± 121.7	0.27 ± 0.045
NTA × C	0.77 ± 0.309	576 ± 34.8	0.22 ± 0.030
TA × P	1.09 ± 0.125	577 ± 79.0	0.23 ± 0.048
TA × C	1.07 ± 0.136	636 ± 66.4	0.22 ± 0.014
P-value	ns	ns	ns

Values represent the means (n = 4) ± SD. Two-way ANOVA *P*-values are reported in the table (ns = not significant). DW = dry weight, SLA = specific leaf area.

Table S2 Nutrient concentrations (g kg^{-1} DW) measured in *I. walleriana* roots at the end of the experiment.

Source of variation	TKN	P-PO$_4$	K	Ca	Mg
Treatment (T)					
No *T. atroviride* (NTA)	14.8 ± 0.58	3.5 ± 0.36	11.8 ± 1.27	16.8 ± 0.85	3.6 ± 0.38
T. atroviride (TA)	14.9 ± 0.75	3.9 ± 0.33	14.0 ± 1.45	17.8 ± 0.77	3.8 ± 025
P-value	ns	ns	*	ns	ns
Substrate (S)					
Peat (P)	15.1 ± 0.83	3.7 ± 0.24	13.1 ± 1.66	17.2 ± 0.80	3.5 ± 0.28
Coir (C)	14.7 ± 0.36	3.8 ± 0.51	12.7 ± 1.92	17.5 ± 1.08	3.9 ± 0.25
P-value	ns	ns	ns	ns	*
T × S					
NTA × P	15.1 ± 0.72	3.5 ± 0.09	12.5 ± 1.31	16.9 ± 0.98	3.4 ± 0.33
NTA × C	14.6 ± 0.23	3.6 ± 0.54	11.2 ± 1.05	16.7 ± 0.89	3.8 ± 0.35
TA × P	15.0 ± 1.04	3.8 ± 0.22	13.7 ± 2.01	17.4 ± 0.71	3.6 ± 0.20
TA × C	14.8 ± 0.48	4.0 ± 0.42	14.3 ± 1.01	18.2 ± 0.70	4.0 ± 0.01
P-value	ns	ns	ns	ns	ns

Values represent the means (n = 4) ± SD. Two-way ANOVA *P*-values are reported in the table (* $P < 0.05$; ns = not significant). TKN = total Kjeldahl nitrogen.
